# Recycling of inorganic waste in monolithic and cellular glass‐based materials for structural and functional applications

**DOI:** 10.1002/jctb.4982

**Published:** 2016-04-13

**Authors:** Acacio Rincón, Mauro Marangoni, Suna Cetin, Enrico Bernardo

**Affiliations:** ^1^ Department of Industrial EngineeringUniversity of PadovaItaly; ^2^ Department of CeramicUniversity of CukurovaTurkey

**Keywords:** Waste Treatment and Waste Minimisation, Green Engineering/Products, Environmental Remediation, Process Intensification

## Abstract

The stabilization of inorganic waste of various nature and origin, in glasses, has been a key strategy for environmental protection for the last decades. When properly formulated, glasses may retain many inorganic contaminants permanently, but it must be acknowledged that some criticism remains, mainly concerning costs and energy use. As a consequence, the sustainability of vitrification largely relies on the conversion of waste glasses into new, usable and marketable glass‐based materials, in the form of monolithic and cellular glass‐ceramics. The effective conversion in turn depends on the simultaneous control of both starting materials and manufacturing processes. While silica‐rich waste favours the obtainment of glass, iron‐rich wastes affect the functionalities, influencing the porosity in cellular glass‐based materials as well as catalytic, magnetic, optical and electrical properties. Engineered formulations may lead to important reductions of processing times and temperatures, in the transformation of waste‐derived glasses into glass‐ceramics, or even bring interesting shortcuts. Direct sintering of wastes, combined with recycled glasses, as an example, has been proven as a valid low‐cost alternative for glass‐ceramic manufacturing, for wastes with limited hazardousness. The present paper is aimed at providing an up‐to‐date overview of the correlation between formulations, manufacturing technologies and properties of most recent waste‐derived, glass‐based materials. © 2016 The Authors. Journal of Chemical Technology & Biotechnology published by John Wiley & Sons Ltd on behalf of Society of Chemical Industry.

## Introduction

In general we refer to organic or inorganic waste, with each category including both hazardous and non‐hazardous waste. Organic wastes are currently destroyed with very high efficiency, even in the case of hazardous substances such as pesticides, polychlorinated biphenyls (PCBs) and persistent organic pollutants (POPs), by incineration.^1^ The handling of inorganic waste (especially if hazardous), on the contrary, is still subject to some controversy, given the heterogeneity of waste streams and the availability of different technologies.

Hazardous inorganic waste derives mostly from metallurgical industrial processes, but can come also from the demolition of buildings and civil infrastructures (realized, for example, with asbestos‐containing cements), or from combustion processes, particularly from municipal solid waste (MSW) incineration. Any form of management, from landfill disposal to recovery, implies a stabilization step applied through several chemical and physical processes, among which vitrification may be treated as the most effective, considering its application even to radioactive waste (the ‘ultimate’ form of hazardous inorganic waste).^2^

Vitrification generally consists of the dissolution of the components of hazardous waste in molten glass, at high temperatures; the components are then incorporated homogeneously into the vitreous structure following the cooling of the melt. This is due to the fundamental characteristics of glass, when properly formulated, such as the high chemical stability and the possibility to contain a huge variety of oxides.^2^, ^3^ Mixing with minerals or already formed glasses (e.g. recycled glasses) is generally performed, if the composition of waste does not contain enough silica, essential for glass formation and chemical stability (low‐silica glasses can be formed, but they may exhibit very poor durability). In some cases the stabilization does not rely on the dissolution of waste, but simply on the thermal destruction, associated with the high temperatures required by glass processing: as an example, asbestos‐containing waste does not contain significant traces of heavy metal oxides, with vitrification recommended essentially for the dismantling of the characteristic, and highly hazardous, fibrous structure.^2^

The main advantages of vitrification can be summarized as follows:

1. flexibility of the process, which allows treatment of many types of waste, such as sludge, contaminated soil, ash, slag from hazardous processing, wet and dry solids in large and variable proportions;
2. destruction of all organics (including the most toxic substances such as dioxins and furans) with an efficiency exceeding 99.99%;
3. excellent stabilization of hazardous inorganic substances (such as heavy metals, radioactive elements, etc.) within the glassy network in ionic form; consequently, low environmental impact and possibility of landfill disposal without any problem, because any inorganic contaminant is retained permanently (any leakage of contaminants is so slow that no detectable adverse environmental effects are produced);
4. substantial reduction in volume of the treated waste (from 20 to 97%, depending on the type of waste);
5. good mechanical and thermal properties of the vitreous product.

**Figure 1:**
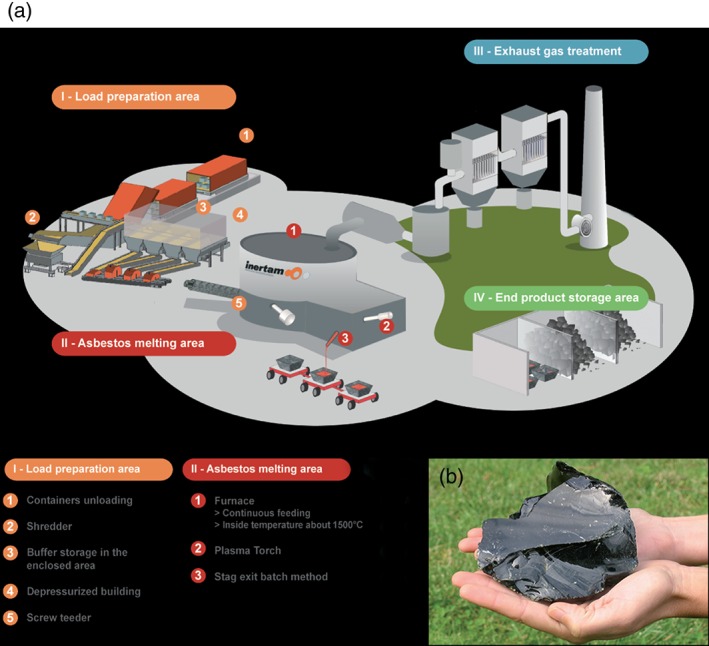
(a) Scheme of the plasma vitrification facility installed by Inertam (Europlasma Group) in Morcenx (Landes, France); (b) block of vitrified asbestos‐containing waste (figures courtesy of F. Protti, Europlasma).

The advantages of vitrification are somewhat compensated by significant drawbacks, such as the high cost of plants and the energy consumption.^2^, ^3^, ^4^, ^5^ The overall sustainability of the process is quite disputable, if the economic advantage relies only on avoided disposal costs. The previously mentioned asbestos‐containing materials, for their intrinsically high hazardousness, justify the adoption even of the most expensive technologies, such as plasma heating, as shown in Fig. 1.^6^ According to Gomez *et al.*,^7^ a transferred arc plasma furnace operating at 1600 °C, in a negative pressure tented enclosure, could be applied to a variety of asbestos‐containing materials (not only a specific type), with successful destruction of all the asbestos polymorphs and conversion into monoliths comprising a gehlenite‐akermanite solid solution. Other wastes, on the contrary, are less likely to be vitrified, with a much less favorable cost/benefit balance, even in the perspective of rising landfilling costs (for the exhaustion of available landfill sites and the hostility of the people towards the opening of new ones). Fruergaard *et al.*,^8^ as an example, applied a life‐cycle assessment (LCA) analysis to several scenarios for the treatment of air pollution control (APC) residues from incineration of municipal solid waste and found that vitrification, followed by landfilling, compared poorly with other management options (including direct landfilling without treatment, backfilling in salt mines, neutralization of waste acid, use as filler material in asphalt, etc.). The issues are represented by the environmental load with regard to GW (global warming potential), in turn greatly affected by the energy consumption (in the order of 700 kWh el per tonne of APC residue), and also by the HTw and HTs (human toxicity potential via water and via soil, respectively), due to air emissions especially of Sb, Hg and As associated with the thermal process. A higher energy efficiency and a good stabilization of pollutant was claimed by Park *et al.*,^9^ who reported the vitrification of incinerator residues by a special combustion furnace, using Brown's gas (a stoichiometric hydrogen/oxygen mixture supplied by water electrolysis), but this has not been confirmed by further literature. Concerning plasma heating, Sobiecka and Szymanski^10^ found that the processing temperature and energy consumption could be decreased significantly, passing from the vitrification of municipal solid waste incinerator fly ash to engineered mixtures of fly ash and chromium‐rich sewage sludge (CRSS); the content of CRSS, however, must be controlled, since the secondary waste may impair the chemical stability of the vitrified mass.

The cost/benefits balance may be much more favorable if the glass produced could provide extra revenue, by fabrication of high‐value products. More precisely, any vitrification approach should be evaluated in the light of a complex economic balance: according to Gomez et al. 7 specifically discussing plasma heating, the avoidance of landfill charges, the added value of the reuse of the vitrified product, the energy production from syngas and the recovery of metals together improve the commercial viability of the process. The vitrified product is the key output of the thermal process, being less sensitive to particular conditions (syngas is a combustible gas from transformation of organics, by pyrolysis, i.e. by thermal treatment in non‐oxidative atmosphere, recognized as a more environmentally friendly technology than incineration due to the higher energy recovery efficiency;^11^ molten metals may separate, at the bottom of glass furnaces, under reducing conditions^3^).

High‐value products can be roughly divided into ‘not glass‐based’ and ‘glass‐based’. ‘Not glass‐based’ products are generally traditional ceramics, such as clay bricks and porcelain stoneware tiles, in which waste glass is used to ‘dilute’ the conventional raw materials;^3^ secondary options are represented by systems in which waste glasses are used as ‘inert’ components, such concretes^12^ and bituminous mixtures.^13^ The use of waste‐derived glass in a mass market application is highly attractive, since it enables safe disposal of a large quantity of waste, but the waste glass contributes only to a limited extent to the final composition of the material and the economic benefit is simply due to the saving of natural raw materials. ‘Glass‐based’ products, in contrast, refer to waste glasses as the dominant component; they are not included in a mass market, but their value may be significantly higher than that of traditional ceramics, owing to particular functionalities, in turn connected to the nature of the waste glass adopted. Common ‘glass‐based’ products are (mostly) monolithic glass‐ceramics, to be used in structural applications, as an alternative to natural stones or ceramic tiles, or glass foams (e.g. cellular glasses), to be used for thermal and acoustic insulation, as reported in a vast literature, including some review papers.^3^, ^14^, ^15^

The manufacturing of both main classes of glass‐based products actually depends on the application of a secondary thermal treatment, implying extra costs. Again, the cost/benefits balance may be adjusted favorably, typically by: (i) engineering the thermal treatment (e.g. reducing the costs of conversion of glass into glass‐ceramics); (ii) obtaining glass‐based products even avoiding preliminary vitrification, starting from engineered mixtures of inorganic wastes, comprising recycled glasses. Inorganic wastes, in this case, are (at least) partially dissolved in the liquid phase offered by the softening of the glass component, undergoing viscous flow sintering. The products evidently lack homogeneity, compared with those from melting, but they may be convenient for the stabilization of wastes with limited hazardousness.^3^, ^14^

**Figure 2:**
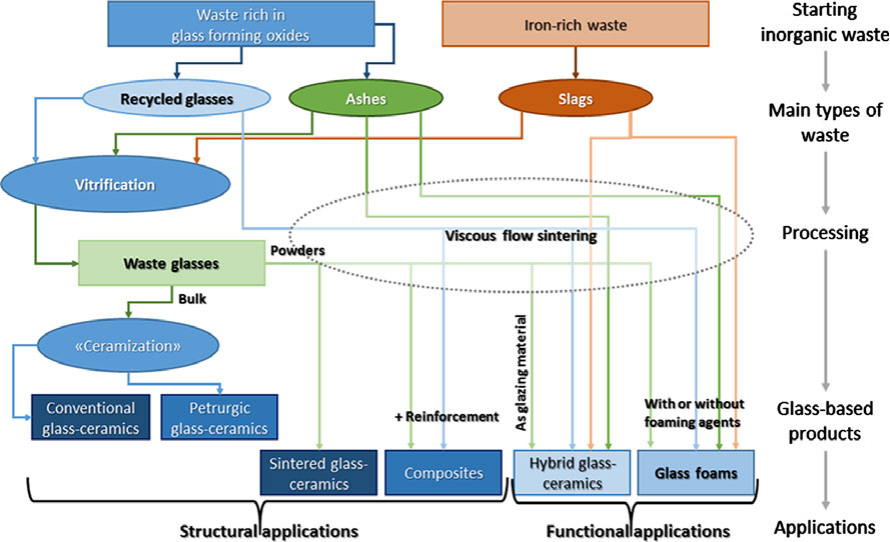
Overview of the succession of topics in the present paper.

The present paper aims at providing an up‐to‐date overview of the technology of glass‐based products as an effective solution for the management of inorganic waste (referring mostly to the literature published after the previously mentioned reviews by Colombo et al. and by Rawlings et al. 3, 14), with special attention to the connection between processing and both structural and functional properties (not focusing only on a specific kind of waste and functional properties, contrary to the review by Chinnam et al. 15). Figure 2 shows a scheme of the methodology used.

## Inorganic waste as raw material for glass‐based products

Generally speaking, inorganic residues, with regard to conversion into glass‐based products, can be divided on the basis of the content of glass formers, notably silica. In fact, silica‐rich waste materials can be vitrified by themselves or by addition of limited quantities of additives, or lead to glass‐based articles, directly by viscous flow sintering, as in the case of recycled glasses. Silica‐poor wastes, on the contrary, cannot lead to glass‐ceramics or glass‐foams, by themselves, either by vitrification and secondary processing or by direct sintering; their use may compromise the overall economic sustainability (the stabilization is due to the use of significant amounts of additives), but we should consider the impact on functionalities of oxides present in these wastes, particularly in the case of iron oxides. The following paragraphs are intended to provide a short overview of the main categories of wastes.

### Wastes rich in glass‐forming oxides

The most interesting silica‐rich waste is actually represented by recycled glasses, as summarized in Table 1. In fact, the term ‘recycled’ is often misleading. Any glass is nominally 100% recyclable, e.g. scrap glass of any composition could be remelted and used for manufacture of the original articles, in a condition of ‘closed loop recycling’. Although recommended for limiting the consumption of energy and natural raw materials, the use of scrap glass in manufacturing new glass articles is possible only after an expensive sorting step, aimed at separation of glass from other materials, like metallic or ceramic contaminants;^16^ the imbalance between the supply of and demand for coloured cullet is also an issue (Butler and Hooper, as an example, specified in their study on glass recycling in the UK that glass manufacturers focus their production on clear glass, while the main cullet supply is heavily influenced by the presence of coloured imported wine and beer containers^17^ ).

**Table 1:** Typical chemical compositions of selected waste glasses (wt%)

Oxide	Soda lime glass^7^	Borosilicate glass^16^	Cathode ray tubes^10^, ^11^		LCD glass^14^	Fluorescent lamps^12^
			Panel	Funnel		
SiO_2_	70.8	72	57.87‐60.7	51.5‐54.1	61.20	67.9
Al_2_O_3_	2.4	7	1.7‐3.76	1.80‐3.21	16.3	2.26
Na_2_O	13	6	7.5‐12.89	6.20‐10.21	‐	17.5
K_2_O	1.1	2	6.9‐7.29	8.2‐9.47	‐	1.6
CaO	9.4	1	0.10	3.5‐3.77	1.5	5.09
MgO	2.1	‐	‐	1.43	1.16	2.96
BaO	0.2	<0.1	7.95‐9.90	0.8‐1.28		0.94
Fe_2_O_3_	0.3	‐	0.22	0.13	B.D.	0.08
MnO		‐	‐	‐	‐	‐
B_2_O_3_	0.12	12	‐	‐	10.72	‐
PbO	0.07	‐	0.01‐0.02	18.40‐22.00	‐	0.79
ZnO	0.12	‐	0.63	0.41	‐	‐
SrO	‐	‐	8.06	0.7‐0.89	‐	
As_2_O_3_	0.02	‐	‐	‐	‐	‐
Sb_2_O_3_	0.01	‐	‐	‐	‐	0.08
Cr_2_O_3_		‐	‐	‐	‐	

Only a fraction of carefully ‘purified’ glass can actually be used for closed loop recycling, with negative effects on the overall sustainability of the same starting glass articles. According to the LCA model by Vellini and Salvioli,^18^ glass containers can be more environmentally benign than PET containers only if the reuse and recycle factors are higher than a certain threshold (e.g. the glass container production scenario with an 80% reuse factor yields better performances than PET container production, whereas a scenario with a 25% reuse factor fails to do so). It is not surprising, as a consequence, that glass cullet should be considered also is in a condition of ‘open loop recycling’, i.e. re‐use in articles different from the original ones, also termed ‘downcycling’,^19^ starting from the production of traditional ceramics.^16^

For common soda‐lime glass a significant fraction enriched in contaminants remains practically unemployed, and is mostly landfilled.^16^, ^20^ The ‘useless’ fraction is obviously more significant for glasses derived from articles that are no longer produced or from articles employing by themselves a limited quantity of recycled materials,^21^ such as glasses from the dismantling of cathode ray tubes (CRTs),^22^, ^23^ lamps (bulbs, fluorescent lamps),^24^ liquid crystal and plasma displays,^25^, ^26^, ^27^ pharmaceutical containers.^28^ ‘Unemployed recycled glass’ can be effectively referred to as ‘waste glass’ (whereas ‘waste‐derived glass’ is the product of vitrification of wastes, that may include unemployed recycled glass). The manufacturing of new glass‐based materials may be seen as the ultimate opportunity for open‐loop recycling and has an undoubted environmental benefit compared with landfilling, as confirmed by recent LCA studies. Meylan *et al.* 19 assessed several scenarios of Swiss waste glass‐packaging disposal and found that the local production of glass foams, for thermal and acoustic insulation, is not only an environmentally sound disposal option (compared even with the production of extruded polystyrene, widely used for the same applications), but it also buffers gross value added losses, in case domestic recycling (and thus glass‐packaging production in Switzerland) ceases in the future. Rocchetti and Beolchini, as a second example, recently showed the sustainability of several open‐loop recycling technologies for CRT glasses.^29^

Ashes from different combustion processes represent the second fundamental example of silica‐rich waste.^30^, ^31^ Coal fly ashes from thermal power plants vary in their composition as a function of the type of coal used, the combustion conditions or the provenance, as shown in Table 2.^14^ Molten coal fly ashes may form glass directly^32^, ^33^ but more commonly some additional oxides are added to lower the viscosity, from minerals^34^, ^35^, ^36^ or from glass cullet.^37^, ^38^ The introduction of nucleating agents such as TiO_2_ or Cr_2_O_3_ to achieve easy transformation to glass ceramics has also been reported.^39^, ^40^

**Table 2:** Typical chemical compositions of selected ashes and slags (wt%)

Oxide	SiO_2_	Al_2_O_3_	CaO	MgO	Fe_2_O_3_	Na_2_O	K_2_O	SO_3_	P_2_O_5_	Cl^−^	Cr_2_O_3_	ZnO
Coal fly ashes^17^, ^21^	18.1‐75.6	7.6‐55.5	0.8‐37.8	3.5‐9.0	3.1‐9.9	0.2‐2.6	0.6‐2.4	2.5‐18.2	‐	‐	‐	‐
MSWI fly ash^31^, ^34^	7.3‐27.5	3.2‐11.0	16.6‐19.5	2.6‐3.1	1.4‐5.0	13.1	11.2	9.8	1.7	10.3‐22.0	‐	‐
MSWI bottom ash^28^	30.3‐47.4	9.9‐13.0	18.8‐23.1	2.8‐2.9	4.3‐10.2	1.9‐4.5	0.9‐1.0	‐	1.2‐1.9	‐	‐	‐
MBM ash^48^	2.3	0.2	46.4	1.3	8.7	8.7	3.5	3.6	34	‐	‐	‐
Sewage ash^42^	39.5	17.2	7.2	2.1	11.1	1.2	2.7	1.9	7.6	‐	‐	‐
Oil shale ash^47^	31.9‐34.7	9.1‐9.4	27.6‐27.7	3.4‐5.9	3.8‐4.4	0.2‐0.3	4.2‐7.4	‐	‐	‐	‐	‐
Rice husk ash^41^	90.7	0.06	1.2	0.8	0.3	‐	1.6	1.6	3.6	‐	‐	‐
BF slag^54^, ^57^	34.39‐36.97	14.79‐14‐47	26.64‐41.67	6.49‐6.7	0.33‐0.63	0.22‐1.43	0.36‐0.65	‐	‐	‐	‐	‐
BOF slag^58^	10.3‐13.7	1.1‐3.9	38.7‐40.4	7.4‐8.2	11.2‐12.9	‐‐	‐	‐	2.0		‐	‐‐
EAF dust^60^	4.4‐5.94	0.65‐1.48	7.5‐20.69	5.21‐9.6	24.28‐52.82	0.91‐6.62	1.01‐1.76	‐	‐	‐	1.12‐15.85	7.57‐13.80
Cu extraction waste^63^, ^64^	24.87‐24.93	0.88‐0.92	0.69‐0.72	0.36‐0.43	67.68‐67.72	‐	0.46‐0.48	2.16	‐	‐	0.12	2.78‐2.82
Red mud^73^	7.8	17.1	11.7	0.6	44.1	3.2	0.1	‐	‐	‐	‐	‐

Ashes from incineration of municipal solid waste (MSW) should be considered as belonging to two distinct categories: (a) MSW incinerator bottom ashes; (b) MSW incinerator fly ashes. Bottom ashes (consisting of glass, magnetic metals, minerals, synthetic ceramics, paramagnetic metals and unburned organic matter) are known to be poorly hazardous, especially in the form of coarse particles.^41^, ^42^, ^43^ On the contrary, MSWI fly ashes constitute a significant form of hazardous waste, since they contain dioxins or furans, to be destroyed, as well as leachable heavy metals (Cd, Cr, Cu, Pb), to be immobilized.^44^ Some formulations, in addition, feature a quite limited content of silica, with a negative impact on the chemical durability of ‘100% ash‐derived’ glasses or on the temperatures required for melting.^45^ Composition corrections with more properly silica‐rich wastes or pure silica provide a simple and effective solution,^46^, ^47^ with additional advantages, i.e. the possibility to extract low boiling point metals.^44^, ^48^

The high temperatures required by vitrification cause the destruction of many hazardous organic compounds,^2^, ^3^ but gaseous emissions still need attention, especially concerning the presence of chloride salts and volatile heavy metal oxides.^4^ Chlorine has a very limited solubility in glasses^49^ and may lead, if uncontrolled, to the formation of hazardous species in the cooling step and to the corrosion of equipments. A preliminary washing treatment may be applied^50^, ^51^, ^52^, ^53^ in order to remove all the water soluble salts and some heavy metals, but the aqueous by‐product could determine a new disposal problem. A pre‐stabilization with chemical agents, such as NaOH, Na_2_S or phosphates,^54^ may promote the formation of less volatile species. The controlled addition of chlorides (e.g. Mg(Cl)_2_) may be considered, in some cases, as a strategy for the removal of heavy metals (particularly Zn) by formation of low boiling point compounds, leaving a practically Cl‐free ash, easier to reuse.^55^

Ashes may derive from any process for energy recovery, reduction of waste volume and destruction of possible organic pollutants. The ashes produced vary in their composition according the different waste incinerated. Rice husk ash, produced in biomass power plants that use rice husk as fuel, has been used as a silica precursor since it contains around 85–90% of amorphous or crystalline silica depending on the combustion conditions.^56^, ^57^, ^58^ Sewage sludge fly ash,^59^, ^60^, ^61^, ^62^ paper sludge ash,^63^ oil‐shale ash^64^ or meat and bone meal ashes^65^ feature a lower content of silica, but they contain significant amounts of P_2_O_5_ (another glass forming oxide) and Al_2_O_3_ (‘intermediate’ glass forming oxide, when combined with alkali or alkaline earth oxides).

Asbestos waste represents a further example of silica‐rich waste, being basically composed of magnesium silicates. The destruction of the characteristic fibrous structure, which constitutes the main health danger, as written above, generally implies very energetic procedures, such as Joule heating,^66^ microwave irradiation,^67^, ^68^ plasma heating,^69^ with vitrification temperatures well exceeding 1500 °C.

### Iron‐rich wastes

The most significant production of iron‐rich wastes is associated with the iron and steel industry; different final products (cast iron or steel) and different processes reflect in important composition variations. Blast furnace (BF) slag is undoubtedly easier to convert into a glass (in turn further transformed into glass‐ceramics^70^, ^71^, ^72^) than other slags, due to the high contents of silica and alumina, usually accompanied by CaO and, in a lower amount, MgO, as reported in Table 2. However, being a well‐known pozzolanic material^73^, ^74^ BF slag is often reused for not‐glass‐based products, such as concrete and geopolymers. Other slags, such as basic oxygen furnace slag (BOF) and electric arc furnace (EAF) slag, dust from electrostatic precipitators, on the contrary, richer in iron oxides, but poorer in glass‐forming oxides, may find applications after composition correction and vitrification^.^ 75, 76, 77, 78, 79

The most recent research refers to iron‐rich waste from non‐ferrous metallurgy. As an example, obtaining copper from ores generates a slag that contains more than 40 wt% iron, present as non‐magnetic iron silicate.^80^ The slag also includes alumina, silica, calcium oxide etc. and oxides of heavy metals that make this waste hazardous. Due to the limited amount of glass‐forming oxides the copper flotation waste is usually mixed with natural raw materials and other residues to achieve glasses to be further transformed. Karamanov *et al.* 81 used an iron‐rich copper flotation waste (Fe_2_O_3_ exceeding 67 wt%) with the addition of blast furnace slag and glass cullet to increase the silica content, lower the melting temperature of the batch and increase the durability of the final glass‐ceramic obtained. Çoruh *et al.* 82 used a similar approach, adding fly ash and perlite to the copper flotation waste (Fe_2_O_3_ content approaching 70 wt%). Ponsot *et al.* 83 successfully prepared glass‐ceramics by mixing crystalline residues of the copper metallurgy, comprising fayalite (Fe_2_SiO_4_), with recycled borosilicate glass.

The iron content may even be recovered, as recently proposed by Yang *et al.*,^84^ who produced an iron‐poor, light coloured glass‐ceramic, as an effect of melting in reducing conditions (coke added to the waste batch, with results conditioned by the CaO/SiO_2_ ratio).^85^

Zinc hydrometallurgy is another important source of iron‐rich waste, as raw material for glass‐based materials. The process yields solid waste with jarosite and goethite, as major crystalline phases, both containing Fe_2_O_3_ in excess of 50 wt%. The recovery of iron is complicated, due to the substantial traces of other oxides, notably silica and heavy metal oxides. Both iron‐rich hydrometallurgy wastes have been successfully employed in glasses later transformed into marble‐like glass‐ceramics, by adoption of a sinter‐crystallization approach,^86^, ^87^, ^88^, ^89^, ^90^ described later in detail.

A third example of iron‐rich waste, from the primary production of a non‐ferrous metal is that of ‘red mud’, i.e. the residue from the well‐known ‘Bayer process’, applied to bauxite in order to separate pure aluminium hydroxide, in turn exploited to obtain both alumina and aluminium. This residue has a limited quantity of glass‐forming oxides; it was successfully used for both dense and porous glass‐ceramics,^91^ by transformation of waste‐derived glasses (comprising red mud and other waste as raw materials) or by direct sintering.

Finally, we should consider tailings from the extraction of other metal ores. Residues from the extraction of tungsten,^92^ gold,^93^ or rare earth metals^94^ were also used as starting materials in the production of glass and glass ceramics; they feature a high content of glass forming compounds, but as in the previous cases the presence of heavy metals represents an environmental problem. Obviously, this is true for residues from the extraction of the same heavy metals, such as Pb;^83^ the decreasing use of heavy metals in many engineering applications, reduces the availability of ‘new’ waste, but it does not affect the amounts of waste produced in the past and not reused.

## Overview of glass‐based products

### Conventional glass‐ceramic monoliths

Glass‐ceramics represent a vast range of materials obtained by controlled crystallization of a glass of selected composition; the products usually possess outstanding properties, such as high hardness and mechanical strength, a thermal expansion coefficient adjustable over a wide range of values (from negative to more than 12 × 10^−6^ °C^−1^), high refractoriness, high chemical durability and excellent dielectric properties. Almost pore‐free articles, starting from an almost pore‐free parent glasses, are easily achieved, differently from other ceramic systems.^95^

The technology of controlled crystallization has been applied to waste glasses since the early 1960s, soon after the discovery of the very first glass‐ceramics.^95^ As a consequence, the manufacturing of glass‐ceramics must be considered as the most established valorization way for inorganic waste, as supported by an extremely vast literature (an excellent review was provided by Boccaccini and Rawlings),^14^ and by extensive industrial production, under trade names such as ‘Slagsitalls’ and ‘Slagceram’.

Sheeted and pressed Slagsitalls have been produced for the last 50 years, with more than 20 billion square meters used in construction, chemical, mining and other branches of industry. The base glasses for both Slagsitalls and Slagceram products belong to the systems CaO‐Al_2_O_3_‐SiO_2_ (CAS) and CaO‐MgO‐Al_2_O_3_‐SiO_2_ (CMAS) (e.g. for Slagsitalls, SiO_2_ = 50–63 wt%, Al_2_O_3_ = 5–11%, CaO = 23–30%, MgO = 1–12%,^14^, ^95^), and are obtained from slags of ferrous and non‐ferrous metallurgy, ashes and waste from mining and chemical industries, with minor compositional adjustments with glass‐forming oxides.

Quite constant glass compositions may be achieved by adjusting the ratios between different wastes, when variations in the composition of single components occur; in any case, changes in the overall glass composition are tolerated, considering the nature of the crystal phases developed. Calcium silicate (wollastonite, CaO · SiO_2_) and calcium feldspar (anorthite, CaO · Al_2_O_3_ · 2SiO_2_) are generally the main crystal phases, with other silicates and alumino‐silicates (pyroxenes, melilites, i.e. rather complex chain silicates, or gehlenite 2CaO · Al_2_O_3_ · SiO_2_ and its solid solutions), present as secondary phases. Depending on the composition, different ions may be accommodated in the same crystal, by formation of quite complex solid solutions (e.g. pyroxenes expressed by the general formula XY(Si,Al)_2_O_6_, where X = Na^+^, Ca^2+^, Fe^2+^, Mg^2+^, etc. and Y = Mg^2+^, Fe^2+^, Fe^3+^, Al^3+^, Cr^3+^, Ti^4+^ etc.),^96^ and the secondary phases may in some cases replace the main ones and vice versa.^95^ The high percentage of crystals, distributed uniformly in the whole volume, with sizes varying from 0.1 to 1 µm, leads to good mechanical strength and excellent abrasion resistance.

Classical glass‐ceramic technology relies on a double step heat treatment (often termed ‘ceramization’) of a previously formed glass object (shaped into the desired form). The treatment provides the nucleation of crystals within the base glass, favored by the separation of some glass components, known as ‘nucleating agents’ (such as Ag or Au colloids, or oxides like TiO_2_ and ZrO_2_), and the crystal growth. The base glass is heated first to the temperature of maximum nucleation and then to the temperature of maximum crystal growth (slightly higher than the previous one), with a holding time at each temperature, before cooling.

For non‐waste‐derived glasses, the nucleating agents are intentionally added to the formulation of the base glass; a key feature of waste‐derived glasses, on the contrary, is the availability of nucleating agents from the same starting waste stream. Some oxides, in fact, present limited solubility in glasses; dissolved in the base glass, they may easily separate upon ceramization. The most significant example is undoubtedly that of iron oxides. Karamanov and Pelino observed the dependence of crystallization on the ratio Fe^3+^/Fe^2+^.^87^, ^97^ They showed that the crystallization of iron‐rich glasses begins with the separation of small magnetite (Fe_3_O_4_) crystals, but the surface oxidation of Fe^2+^ to Fe^3+^ causes a change in the chemical composition, with the formation of hematite (Fe_2_O_3_), thus decreasing the total amount of crystal phase and changing the reaction order of the crystallization process.

Fe_2_O_3_ is also interesting for its interaction with sulfur: Suzuki *et al.* 62 showed that, due to the presence of Fe_2_O_3_, sulfur and carbon, iron sulfide, FeS, could be formed and favour the precipitation of anorthite. Sulfides also control the colour of glass‐ceramics: in Slagsitalls, the addition of ZnO turns the colour of glass‐ceramics from grayish black, given by FeS or MnS, to white, due to the formation of ZnS (together with FeO or MnO).^95^

### Glass‐ceramic monoliths from alternative routes

The above described nucleation/crystal growth step may be difficult to control and economically expensive. The overall costs may be reduced by application of two distinct processes:

- petrurgic process,
- sinter‐crystallization process.

The petrurgic process resembles the process of crystallization of natural rocks.^98^ In this process (applied since the 1970s, with the development of ‘Silceram’ ceramics from metallurgical slags^14^), crystals nucleate and grow directly upon cooling of the glass melt, with an intermediate temperature holding stage, which can sometimes be avoided. As an example, Francis *et al.* 98 reported the feasibility of crystallization upon controlled cooling (from 1 to 10 °C min^−1^) of glasses obtained from mixtures of coal ash and soda lime glass melted at 1500 °C, without any intermediate step. Nominally, the process is not ‘glass‐ceramic’, since the base material is not available as an actual glass (material below the transition temperature) at any stage, but keeps the concept of finely controlling the microstructure by control of the heat treatment conditions, particularly the cooling rate. More precisely, in the paper by Francis *et al.* faster cooling rates are found to promote magnetite, with samples exhibiting magnetic properties, while slow cooling rates cause the formation of plagioclase and augite.

The sinter‐crystallization process, consisting of the viscous flow sintering of glass frits with concurrent crystallization, is somewhat more refined. Originally applied for the first time during the 1970s, for the manufacturing of the well‐known Japanese ‘Neoparies’ tiles (used in the building industry)^95^ the process has been successfully transferred to the valorization of waste glasses.^99^, ^100^ It specifically provides valid solutions to the usual drawbacks of waste‐derived glass‐ceramics developed by traditional processes, i.e. the control of defects and the visual appearance.

The removal of gas bubbles from the glass melt requires high temperatures and long holding times, i.e. a carefully controlled refining step during vitrification. This operation may be difficult with waste glasses, which are usually dark and feature a low thermal conductivity by radiation, due to the amount of heavy metals, with the risk of leaving many pores in the base glass, later ‘frozen’ by ceramization. Concerning the visual appearance, that of waste‐derived glass‐ceramics is generally rather inferior to that of natural stones and traditional ceramics.^2^, ^3^

**Figure 3:**
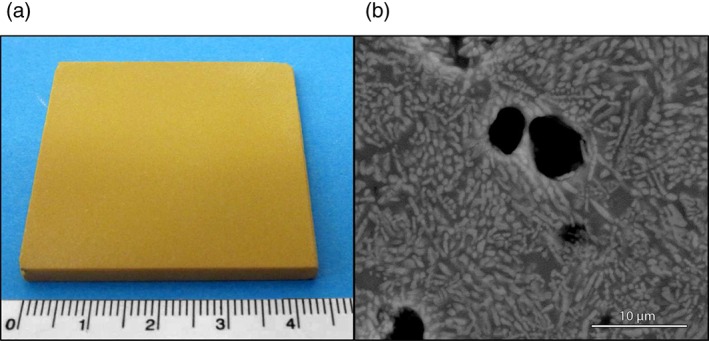
(a) Example of sintered glass‐ceramic tile, obtained by use of powders <100 µm of a CAS glass (from the melting of a basalt rock/boron waste mixtures);^108^ (b) example of microstructure of a sintered glass‐ceramic from a CaO‐MgO‐ZnO‐Al_2_O_3_‐SiO_2_ glass, with evidence of Ca(Mg,Zn)Si_2_O_6_ pyroxene crystals.^108^

When applying the sintering route, there is no need to refine the melt before casting into a frit, thus reducing cost and gaseous emissions. In fact, the vitrification may be conducted in small plants and in particularly short times, favoring the immobilization of components which could vaporize with longer heat treatments. The ground glass powder is subsequently pressed and heated to a certain temperature, at which viscous flow densification occurs together with crystallization. The crystallization, generally starting at the contact points between adjacent glass granules,^100^ gives a pleasant visual appearance to the products (Fig. 3(a)). More significantly, a relatively high degree of crystallization may be achieved in very short times, the surface of glass being a preferred site for nucleation.^101^, ^102^, ^103^, ^104^

In general, ground glass is easier to devitrify than bulk glass with the same composition, so that nucleating agents are not needed. In some cases, the holding time at the sintering temperature may not exceed 30 min, being also accompanied by very fast heating rates (even ‘direct heating’ is possible, that is the direct insertion of glass powder compacts in the furnace directly at the sintering temperature), thus configuring a ‘fast sinter‐crystallization’.^105^ Pyroxenes, wollastonite and anorthite (with solid solutions) are very common crystal phases (Fig. 3(b)). However, the remarkable nucleation activity of fine glass powders (<40 µm) has been found to enable the quite unusual precipitation of alkali feldspars and feldspathoids, such as sanidine and nepheline, as main crystal phases.^106^, ^107^

**Figure 4:**
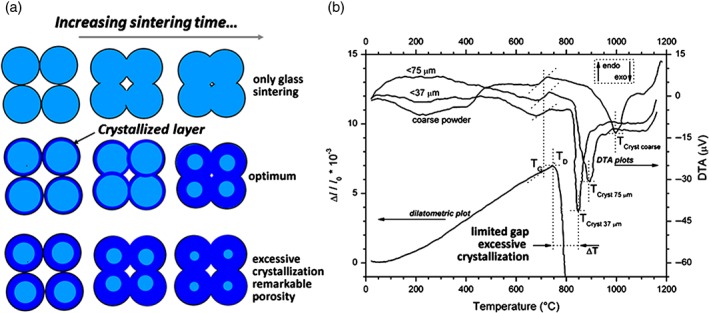
(a) Schematic representation of the sinter‐crystallization process; (b) reduced softening‐crystallization gap for a waste‐derived glass (glass from plasma vitrification of MSWI fly ash^113^).

The sinter‐crystallization process relies on a delicate balance between viscous flow sintering, surface crystallization and even bulk crystallization, i.e. crystallization operated by the separation of components acting as nucleating agents. As shown by Fig. 4(a), if the crystallization at the glass surfaces is too intensive, the densification may be incomplete; on the contrary, for a glass not prone to surface crystallization, the viscous flow sintering predominates, with the formation of a sintered glass body.

The viscous flow/crystallization balance is sensitive to many conditions, e.g. the oxidation state and the heating rate. Starting from an iron‐rich waste glass, Karamanov *et al.* 109 observed that the addition of C (1.5–2%) to the glass batch increased the magnetite phase and enhanced the crystallization rate. Liu *et al.* 110 found that iron oxidation, causing an increase of viscosity, reduced the crystal growth of silicates; this fact could be prevented by applying sintering in an inert (N_2_) or reactive (CO) atmosphere. Bernardo *et al.*,^91^ on the contrary, starting from a base waste glass with a low Fe^2+^/Fe^3+^ ratio, observed that magnetite was promoted by oxidation, more pronounced for fine glass powders (<40 µm) than for coarse ones (<80 µm). Karamanov *et al.* 111, 112 reported that low heating rates favor bulk crystallization, and sintering may be inhibited by the crystal phase, causing incomplete densification, whereas high heating rates favor sintering, with lower crystal phase formation, by reduction of glass surfaces.

It has been shown in many papers^64^, ^91^, ^105^, ^106^, ^107^ that, in the presence of fine glass powders (<40 µm), crystallization may be achieved right at the temperature of the crystallization exothermic peak in the DTA plot of the same powders. More recent investigations,^113^ however, highlighted that optimum crystallization is achievable only if the crystallization peak is located at a temperature suitably higher than that corresponding to the dilatometric softening point, i.e. the temperature at which viscous flow becomes appreciable.^114^ If the temperature difference is limited, as shown in Fig. 4(b) for a glass from the plasma vitrification of MSWI fly ashes, the glass‐ceramics obtained are remarkably porous (as illustrated by the lower part of Fig. 4(a)) and improvements in the densification are achievable only by increasing the sintering temperature and the heating rate.

### Cellular glass‐based materials

Cellular glasses generally offer high surface area, high permeability, low density, low specific heat, high thermal and acoustic insulation and high chemical resistance.^115^ When mostly closed‐celled, they are referred to as ‘foams’.

In most cases, glass‐based foams represent a further variant of glass sintering. If sinter‐crystallized glass‐ceramics depend on a delicate balance between viscous flow sintering and crystallization, glass foams depend on a similarly delicate balance between viscous flow sintering and gas evolution. Crystallization may occur as well, with contasting effects, discussed later.^116^

Gas evolution depends on oxidation or decomposition reactions of additives mixed with glass powders.^115^ Oxidation reactions are in turn associated with the release of CO_x_ gas (carbon monoxide or carbon dioxide) from C‐containing compounds, e.g. carbon black, graphite, SiC, organic substances, reacting with oxygen from the atmosphere. Decomposition reactions are those provided by carbonates (mainly Na‐ and Ca‐carbonates) or sulphates, leading to the release of CO_2_ or SO_x_; a special variant comes from oxides of metals undergoing transition from high to low valence state and releasing oxygen gas (e.g. MnO_2_ being transformed into MnO).^16^, ^115^ Oxidation and decomposition may even overlap, as in the case of nitrides, being transformed into oxides and releasing nitrogen gas.^115^

Considering the difficulty of controlling both foaming and crystallization of waste‐derived glasses, the most suited starting materials for glass‐based foams^16^ are the waste glasses (i.e. as previously discussed, ‘unemployed recycled glasses’) with limited tendency towards crystallization. However, some crystals may form even in this case, owing to secondary reactions involving the glass and the additives. CRT glasses may form wollastonite (calcium silicate, CaSiO_3_) or colloids of metallic lead, when foamed by decomposition of CaCO_3_ 65, or by oxidation of SiC and TiN,^116^, ^117^ due to CaO/glass interaction (CaO + SiO_2(glass)_ → CaSiO_3_) or reduction of PbO (yielding the oxygen necessary for the oxidation of carbides and nitrides). The crystallization may be intentionally stimulated by using glass cullet mixed foaming agents as well as with glasses more prone to devitrification, waste‐derived or not,^118^, ^119^ or directly with inorganic waste, mainly represented by fly ash.

Foaming additives, such as SiC, may be quite expensive and have a negative impact on the overall cost/benefit balance. However, many recent investigations have demonstrated the effectiveness of foaming agents representing by themselves forms of inorganic waste. SiC could derive from the waste originated by the polishing of glass or traditional ceramics, i.e. a mixture of silicate residues (the abraded materials) and SiC (the abrasive medium),^16^ as well as from burned wastes of abrasive papers.^122^ Residues from glass polishing could consist of fine glass powders mixed with oil‐based coolant;^123^ also in this case the oxidation of the additive causes substantial foaming upon sintering. Carbonaceous residues, as foaming agents, may derive from common industrial waste, e.g. sawdust.^124^ A further example is that of boron waste (mining residues from excavation of B‐rich minerals), featuring a remarkable content of CaCO_3_ and leading to foams with a complex distribution of crystal phases, when combined with soda‐lime glass and clay.^65^ ‘Mineral’ CaCO_3_ can be replaced by ‘natural’ CaCO_3_ in the form of egg‐shell waste.^24^ It should be noted, in any case, that the foaming reaction must take place in a pyroplastic mass, determined by the softening of glass powders, with a specified viscosity (in the order of 10^3^–10^5^ Pa · s.^115^). While the decomposition of carbonates well matches with the softening of glasses from dismantled CRTs (known for their low characteristic temperatures),^125^, ^126^, ^127^, ^128^, ^129^, ^130^ it may be more difficult to exploit with other glasses.

**Figure 5:**
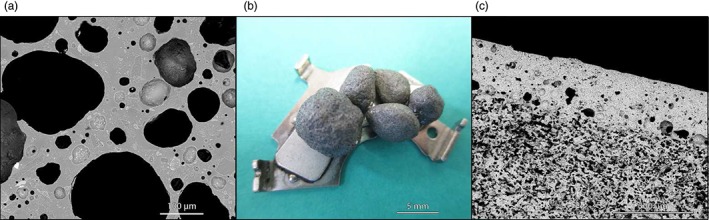
Examples of waste‐derived porous glass‐based materials:^108^ (a) glass foam from cullet/basalt scoria mixture; (b) magnetic glass foam granules from glass cullet/lead metallurgy slag attached to a permanent magnet; and (c) example of layered glass‐ceramic.

Special attention must be given again to iron oxides, in waste‐derived glasses or slags. Fe_2_O_3_ (iron as Fe^3+^) is interesting for its reduction, at moderate temperatures (1000 °C), into FeO (iron as Fe^2+^) with release of oxygen (2 Fe_2_O_3_ → 2 FeO + O_2_).^131^ The release is in turn exploited for foaming, both indirectly and directly. In the first case, the extra oxygen (in addition to that from the atmosphere) from Fe‐rich glasses optimizes the reaction of C‐containing compounds or nitrides, as already done for the industrial process of commercial glass foams (the well‐known Foamglas® by Pittsburgh Corning^132^). In the second case, oxygen is by itself the foaming gas, as found by Appendino *et al.* 133 (condition known as ‘bloating’). The addition of soda‐lime glass to waste‐derived glass powders is currently under investigation in order to control the size and morphology of oxygen bubbles.^108^ A fundamental alternative to iron‐rich waste‐derived glasses is provided by iron‐rich minerals, such as basalt scoria (unemployed volcanic mineral)^134^ (Fig. 5(a)), and metallurgical slags (e.g. slag from the refining of precious metals,^135^ or from lead metallurgy, see Fig. 5(b)). In the latter case, the crystallization is proof of both glass/waste interaction and effectiveness of reduction (with hematite, Fe_2_O_3_, available as major phase from the crystallization of vitreous slag alone, replaced by calcium‐iron silicates, with iron as Fe^2+^, or magnetite, for glass/slag foams).

Substantial crystallization, if negative for the development of highly porous foams, is an advantage for cellular structures, with open‐cell morphology, with a process resembling that of crystalline ceramic foams, for which a three‐dimensional, trabecular structure is templated by polyurethane (PU) sponges. The crystallization of glass, deposited on the PU substrate, prevents collapse by viscous flow. As an alternative, sacrificial materials in the form of polymethylmethacrylate (PMMA) or polyethylene (PE) may be used to template the porosity: if the viscous flow of surrounding glass powder defines the cells, the cellular structure is again stabilized by the crystallization (a silicone resin may help as low temperature binder, before sintering).^65^, ^136^

### Hybrid glass‐based materials

The viscous flow sintering approach can lead to monolithic materials with high Young's modulus, modulus of rupture, hardness and fracture toughness, suitable for structural applications, even avoiding the melting stage (at 1350–1400 °C), by sintering of mixtures comprising waste glasses, at moderate temperatures (generally not exceeding 1000–1100 °C). We can generally divide these materials into: (i) glass matrix composites; (ii) glass‐ceramics from direct sintering; and (iii) hybrid glass‐ceramics, from combinations of the first two classes.

The development of glass matrix composites from waste was pioneered by Boccaccini *et al.* 137 who reported the introduction of up to 20 vol% low‐cost alumina platelets in a glass matrix developed by sintering borosilicate glass cullet mixed with fly ash. The particular reinforcement has been successfully proposed for other waste glasses, such as CRT glasses.^138^ As in the case of glass foams, the additive can be a waste by itself, as shown by Ferraris *et al.*,^139^ who reported the introduction of solid waste from an aluminium foundry. The concept of composites can be transferred also to glass foams, reinforced with particulates (e.g. TiO_2_)^140^ and fibres.^141^, ^142^, ^143^ The foaming may be related to the nature of the reinforcement, operating with metal fibres (Hastelloy X fibres)^144^ mixed with borosilicate glass, under microwave radiation. The fibres tips act as ‘nucleating agents’ for pores, since the higher local electric field strength in their vicinity, connected with their pronounced radius of curvature, enhances the power dissipation in the surrounding glass matrix, which overheats, releasing gasses. The metal fibres prevent cracking and disintegration of the composites during processing and favour the application of the cellular glass‐based composites obtained as lightweight components for electromagnetic interference shielding.

Differently from composites, in which the mechanical properties are conditioned by a secondary phase, physically dispersed in a glass matrix (e.g. the brittleness of glass is reduced owing to crack deflection at glass/reinforcement interfaces or plastic deformation, in the case of metal reinforcement), glass‐ceramics from direct sintering rely on the formation of silicate and alumino‐silicate crystals, similar to those produced by crystallization of waste glasses. This fact supports the use of the term ‘glass‐ceramic’, despite the absence of a vitrification step.^3^, ^14^

In addition to the savings in energy required by the overall manufacturing process,^14^ direct sintering is advantageous for reducing the volatilization of some pollutants (e.g. fluorides);^145^ on the other hand, as previously stated, the products lack homogeneity, so that some pollutants could remain concentrated in some areas of the samples, although the leachability of sintered residues could be in any case lower than that of untreated waste (Zacco *et al.* specifically mention the viability of direct sintering of incinerator residues^44^). Finally, direct sintering can be used also for highly porous glass‐ceramics, with a glass phase originating from part of the waste or from fluxes, such as Na silicate and Na borate.^128^, ^146^, ^147^, ^148^

‘Hybrid’ glass‐ceramics are systems in which many features of the previously presented glass‐based materials are successfully combined. As an example, platelets can be used as reinforcing phase both with waste‐derived glasses unable to crystallize,^149^ for glass matrix composites, as well as with glasses subjected to sinter‐crystallization, for glass‐ceramic matrix composites: Bernardo *et al.* 106 prepared composites with a bending strength of 163 ± 14 MPa and a fracture toughness of 1.9 ± 0.1 MPa m^0.5^, by the addition of up to 15 vol% alumina platelets to a waste glass capable of sinter‐crystallization and leading to a nepheline‐based glass‐ceramic matrix. Appendino *et al.* 133 and Aloisi *et al.* 150 found similar results with a glass from MSW incinerator fly ash mixed with alumina waste.

Layered glass‐ceramics, object of more recent investigations, are even more complex. They refer to a specific market need, in the field of building materials, i.e. that for lightweight tiles, with low water absorption (below 2%, for optimized frost resistance), to be placed vertically. Anchored to metal frames, in turn fixed on main building walls, these tiles constitute the so‐called ventilated façades. The air gap between the tiles and the wall contributes positively to the thermal insulation (minimizing thermal losses, in winter, and minimizing overheating, in summer). A solution may come from traditional porcelain stoneware tiles with an engineered porosity, but they are obtained by using expensive foaming agents (SiC, CeO_2_; the foaming of porcelain stoneware must be matched with sintering, as in glass foams, but at much higher temperatures).^151^ In layered glass‐ceramics, a single‐step treatment causes the direct sintering of a base body formed by a glass/waste mixture and the sinter‐crystallization of a glaze, obtained from a glass in turn derived from the same starting materials.^152^, ^153^ The high residual porosity (in the order of 30–35%), the high water absorption, the poor visual appearance and the limited chemical homogeneity of the base body are not significant issues, since mechanical strength, colour and stabilization of pollutants depend on the much denser glaze (the glazed side is the one to be exposed directly to the environment). Strength, colour and shrinkage of the glaze can be adjusted by using secondary phases and waste glasses. Vitrification of waste is reputed to be sustainable, since it is applied only to a limited amount of starting materials; the single firing reduces the costs associated with the deposition of a glaze.

## Structural and functional properties

### Main properties of waste‐derived glass‐based materials

The replacement of natural stones, such as granite and marble, has been a fundamental aim of waste‐derived glass‐ceramics, since the 1960s, with the first examples of Slagsitalls.^95^ As discussed above, the process conditions (sinter‐crystallization instead of conventional treatments, application of glazes) may provide a solution to the general problem of poor visual appearance or unpleasant colouration of waste‐derived materials, compared with natural stones or high quality traditional ceramics, like porcelain stoneware; the mechanical properties, on the contrary, have always been considered a strength of glass‐ceramics compared with other materials.^154^, ^155^ Many authors claim that high strength materials are associated with the precipitation of very fine silicate and alumino‐silicate crystals; just to cite some examples, Boccaccini *et al.* 156 showed an almost 3‐fold increase of bending strength (from 90 to 240 MPa) and fracture toughness (from 0.6 to 1.7 MPa m^0,5^) for a glass‐ceramic with respect to the parent glass, produced from vitrification of MSW ash; Oveçoglu^157^ produced slag‐based glass‐ceramics with a high bending strength (>300 MPa) and excellent fracture toughness (5.2 MPa m^0.5^); Peng *et al.* 158 demonstrated the feasibility of glass‐ceramics with nano‐sized crystals (<200 nm), from the controlled crystallization of a glass from high alumina coal fly ash. A collection of mechanical data is presented in the review paper by Rawlings *et al.* 14

The strength data may lead to some misunderstandings. Brittle materials are well known for the sensitivity of strength on the dimension of samples; strength data (typically bending strength data), consequently must refer to samples with standardized dimensions. Alternative approaches correspond to the application of Weibull's statistics or to the assumption of a benchmark. In the first case, strength data of laboratory scale samples can be converted into strength data for samples of standardized dimensions, by means of scaling equations based on Weibull's modulus,^159^ as done by Bernardo *et al.* 60 for glass‐ceramics from vitrified sewage sludge pyrolysis residues, compared with traditional ceramics. In the second case, waste‐derived glass‐ceramics may be compared with traditional ceramics (the benchmark) using samples of the same dimensions.^160^

**Figure 6:**
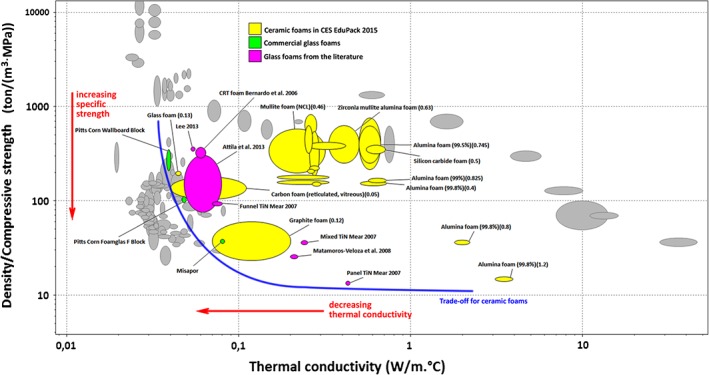
Specific strength‐thermal conductivity trade‐off plot for foams (non‐ceramic foams are shown in grey colour) ‐ figure and basic data derived from CES EduPack 2015 program package;^162^ extra data inferred from the literature.^123^, ^125^, ^163^, ^164^, ^165^

The mechanical properties of highly porous glasses and glass‐ceramics are generally expressed in terms of compressive strength, practically not sensitive to the dimension of samples (provided that the dimensions of test samples are adequately bigger than pore size, and buckling is avoided),^161^ but they must be discussed in the light of the main applications, in the field of thermal and acoustic insulation. Figure 6 demonstrates that glass‐based cellular materials can be considered as ‘thermo‐structural materials’ for their distinctive combination of thermal properties and strength.

The thermal conductivity can be lowered (solution placed at the left of the trade‐off curve) only operating with less stable polymeric foams; in fact, contrary to polymeric cellular materials, glass‐based cellular materials are non‐flammable and flame resistant, chemically inert and not toxic (even if waste‐derived), rodent and insect resistant, bacteria resistant, water and vapour resistant.^115^ In selected cases, the thermal conductivity of glass‐based cellular materials may be particularly low, in the order of 0.05 W mK^−1^. Among stable, inorganic cellular materials (coloured ‘bubbles’ in Fig. 6), glass foams express the best compromise between low thermal conductivity and high specific strength (ratio between compressive strength and density, of vital importance for lightweight components). As an example, ‘Misapor’ foams (commercial glass foams from recycled soda‐lime glass foamed with SiC additive)^163^ represent a ‘non‐dominated’ solution: with the same thermal conductivity, no material exists with a higher specific strength (or lower density/compressive strength ratio); with the same specific strength, no material exists with a lower thermal conductivity.

Most ceramic foams (in yellow in Fig. 6) are quite far from the trade‐off curve; the superiority of glass foams can be justified on the basis of the distinctive closed‐cell morphology. In general, the crushing strength of a cellular material derives from the bending strength of the solid, with a scaling factor in turn associated with the relative density (*ρ_r_*, ratio between density of the porous body and density of the solid phase, or ‘true density’), according to the equation (derived from the well‐known Ashby model^161^):

$$\frac{\sigma_{cr}}{\sigma_{fs}}\approx 0.2{\left(\phi \rho_{r}\right)}^{3/2}+\left(1-\phi \right)\rho_{r}$$

where *ϕ* defines the fraction of solid at the cell edges (1–*ϕ* obviously stands for the fraction of solid at the cell faces), *σ_cr_* is the compressive strength and *σ_fs_* is the bending strength of the solid. A closed‐cell morphology corresponds to significant contribution from the linear term, absent for open‐celled foams (or ‘sponges’, with *ϕ* = 1). Any glass‐based cellular material, however, is not ‘ideally closed‐celled’ in its mechanical behaviour; in fact, if closed walls between adjacent pores are in turn porous, their contribution to the strength is quite poor, despite the positive contribution to the minimization of thermal insulation; in addition, as brittle foams, porous glasses are subjected to a size effect, so that beyond differences in the distribution of solid phase between cell and faces (*ϕ* ratio), the strut strength increases with decreasing cell size; finally, pores without uniform shape and dimensions lead to low strength, as an effect of non homogenous stress distribution (and local stress concentration).^125^

The effect of partial crystallization (in glass‐ceramic foams) on crushing strength is not straightforward. On one hand, it increases the bending strength of the solid phase (high strength foams are actually partially crystallized;^118^, ^119^) on the other hand, the crystallization may strongly increase the apparent viscosity of the glass, limiting foaming and hindering the formation of well‐defined, solid walls. In other words, potential improvements may be counterbalanced by the weakening effect of inhomogeneous microstructures.^116^ Surface nucleation is even enhanced with a porous body, owing to the higher specific surface: Bernardo^127^ has shown that a foam from a given waste‐derived glass reached the same crystallization degree in 1 h as a monolith in 2 h at 880 °C.

### Magnetic properties

Iron‐rich ceramic phases, such as magnetite and other ferrites (oxides with the general formula M^2+^Fe_2_O_4_, or M^2+^O · Fe_2_O_3_; magnetite, Fe_3_O_4_, may be expressed as FeO · Fe_2_O_3_) are well known for their ferrimagnetic behaviour.^166^ Considering the great availability of iron in waste‐derived glasses, and the limited solubility of iron oxides in glasses, as stated above, it is not surprising to find ferrimagnetic phases in waste‐derived glass‐ceramics. The magnetic behaviour achieved may be tuned by changing composition, processing temperature, annealing time, particle size (for frit‐derived glass‐ceramics), heating and cooling rates.

Romero *et al.* 99 from a glass derived from the combination of goethite, dolomite and soda‐lime glass cullet, found that the magnetic properties are directly correlated with the iron oxide: low concentrations (15.6–18 wt%) lead to a paramagnetic behaviour, with iron ions distributed in solid solutions, whereas high concentrations (18.4–25.8 wt%), exceeding the solubility limit, lead to the precipitation of magnetite particles (Fe_3_O_4_). The magnetic clusters obtained provide a superparamagnetic behaviour, but the behaviour may turn into ferrimagnetic when the precipitates are close enough to exhibit fully magnetic behaviour (iron oxide content above 22.9 wt%).

Min'ko *et al.* 167 studied the separation of magnetite in more detail. The crystallization of magnetite particles may take place at relatively low temperature, starting from 700–800 °C; higher temperatures favour the formation of non‐magnetic species and the magnetic susceptibility decreases. This was confirmed by Francis,^168^ who studied the annealing of a glass, from the melting of furnace slag and flue dust, for 2 h at 800 to 1000 °C, and found that saturation magnetization decreases as a consequence of the transformation of the magnetic species (magnetite or γ‐maghemite) into non‐magnetic phases. The magnetic susceptibility is also maximized for smaller particle sizes: as an example, Lorenzi *et al.*,^78^ who used dust from an electrostatic precipitator as iron source (combined with glass cullet), obtained a ferromagnetic material after direct casting of the melt and explained the increase of saturation magnetization in terms of higher content of ferrimagnetic species (magnetite/maghemite) and a peculiar size distribution of the particles (nanometric or micrometric crystals) within the samples.

The energy losses associated with hysteresis cycles of ferro‐ and ferrimagnetic materials may lead to substantial heating of samples under alternating magnetic field, as widely shown for iron‐containing biocompatible glass‐ceramics, generally exploited for hyperthermia cancer therapy.^169^ The concept of indirect heating has been applied even to iron‐rich waste‐derived glass‐ceramics, for the same medical application or not. In fact, Abbasi *et al.* 170 obtained a biocompatible glass‐ceramic material from the direct sintering of soda‐lime–silica waste glass and strontium hexaferrite particles: with an optimized hexaferrite content of 20 wt% the energy loss could exceed 75000 erg g^−1^, in agreement with the requirements of hypothermia therapy. As an alternative, Ponsot *et al.*,^83^ obtained ferrimagnetic glass‐ceramics from the sintering of borosilicate waste glass with iron‐rich slags (from copper and lead metallurgy), reaching high temperatures (exceeding 300 °C) after the application of an alternating magnetic field for very limited times (60 s), that could be good candidates for innovative heating elements (e.g. in cooking tops), considering the resistance to thermal shock (no cracks are developed upon sudden cooling, as an effect of the low expansion coefficient of the borosilicate glass matrix) and the chemical stability (confirmed by cytotoxicity studies). Further applications, e.g. in the field of electromagnetic shielding (the energy losses associated with magnetization hysteresis could be exploited to reduce the intensity of low frequency electromagnetic waves^171^), are in progress.

### Electrical properties

Like most glasses, waste‐derived glasses exhibit low electronic conductivity; the overall electric conductivity and the polarizability, however, are conditioned by the ionic mobility.^172^ Saccani *et al.* 173 studied the electrical behaviour of different glasses derived from the melting of municipal solid waste incinerator grate ash and soda‐lime cullet and observed that with increasing content of incinerator waste the electrical conductivity decreased (conductivity below 10^−14^ S cm^−1^ at room temperatures), as a consequence of increased content of alkaline‐earth ions, strengthening the silicate network and hindering the motion of alkali metal ions. This is accompanied by low values of dielectric permittivity and loss factor as well as by significant chemical durability, so that the materials developed could be a valid alternative to E glass (for fibres) for insulators. Similar results were described by Elalaily *et al.*,^174^ who reported a conductivity of about 10^−8^ S cm^−1^ (at room temperatures) for glasses derived from blast furnace slag, that could be increased significantly by γ‐irradiation, as a consequence of an increasing number of vacancies and vacancy interstitials recombining or migrating to the glass surface.

The multiple valence states of iron (Fe^2+^, Fe^3+^) represent a fundamental factor for the conductivity of waste‐derived glasses, as evidenced by Min'ko *et al.*,^175^ on glasses prepared using mining overburden (sand, chalk, marl and crystalline shale) and ore‐dressing wastes of iron quartzite. A wide range of electrical resistivity (10^11^–10^14^ Ω cm) may be achieved, depending on the ratio Fe^3+^/Fe^2+^ (Fe^3+^ acts as glass former, so that it favours high resistivity). In a more recent study^167^ the same authors, using the same raw materials, showed that crystallization had a dramatic impact on electrical properties, passing from magnetite to pyroxene with increasing annealing temperature. A maximum conductivity of 4.1 × 10^−5^ S m^−1^ was detected at 800 °C, in the presence of magnetite precipitates.

Lorenzi *et al.* 78 recently reported that iron‐rich glasses may lead to glass‐ceramics with low electrical resistivity, in the order of 20 Ω m, attributed to the fact that iron oxide nanoclusters, when their density exceeds a certain threshold value, can give rise to percolation effects that can strongly reduce the resistivity of the material, up to values that make it suitable for applications in antistatic surfaces.

### Other properties

A high infrared radiance glass‐ceramic was obtained by Wang *et al.* 176 using coal fly ash and titanium slag with MgCO_3_ additives. They studied the nucleation and the crystallization process and concluded that enhanced infrared radiance performance was achieved due the iron impurities of the initial materials, leading to the formation of iron‐substituted cordierite (Mg,Fe)_2_Al_4_Si_5_O_18_. The specific phase, even non‐substituted, is effectively interesting for its emissivity,^177^ which could be exploited for panels placed in building façades heavily exposed to the sun, in order to minimize the absorbed heat (at least a small fraction of radiation, in an opaque material, is not reflected) and the consequent so‐called ‘heating island effect’.^178^

An open‐celled glass foam (open porosity exceeding 70%), coated with TiO_2_, prepared by Lebullenger *et al.* 179 using glass waste from the automobile industry mixed with AlN, recently exhibited significant potential for photo‐catalysis. In particular, foam glasses with specific coatings may present photocatalytic activity in the UV region and can be used for toluene decomposition in the gas phase, as an alternative to cellulose/titania commercial supports, with the fundamental advantage of being more easily reusable (the restoration of photocatalysis power, by heat treatment or any other cleaning process, is obviously more difficult to realize with an organic support).

Additional catalytic supports were developed by Dominguez *et al.*,^180^ who developed reticulated ceramic foams by replication of sacrificial PU templates with slurries comprising waste glass, dust and reduction slag from stainless steel production and Portland cement. The catalytic activity (particularly for the CO oxidation reaction) was due to the application of coatings consisting of Al_2_O_3_, CeO_2_ and gold, but the metal content of the wastes was found to have a positive influence on the activity of the foams. Glass‐ceramic foams, developed in a similar way and featuring the separation of iron oxide phases, considering the well‐known activity of these compounds (particularly in thermochemical water splitting,^181^) could constitute an interesting extension of the approach.

Highly porous materials from the sintering of glass mixed with as received or weathered volcanic ash may constitute valid humidity control devices, as shown by Vu *et al.* 182 The characteristic low temperatures (not exceeding 820 °C) required by viscous flow sintering of glass make it a good ‘glue’ for minerals from volcanic ash. Hydrated alumino‐silicates with distinctive moisture retention (mordenite and allophane) did not decompose completely, keeping a substantial micro‐porosity, despite infiltration of softened glass. As an example, a mixture comprising 30 wt% weathered volcanic ash and 70 wt% waste glass, sintered at 800 °C, with a holding time of only 5 min, led to ceramics with a BET surface area and porosity exceeding 160 m^2^ g^−1^ and 50%, respectively, with a pore size of approximately 9 nm in diameter.

## Concluding remarks

The continuously increasing production of hazardous and toxic wastes, as well the lack of solutions for less problematic wastes, such as unemployed glasses, undoubtedly favour the manufacturing of both monolithic and cellular glass‐based materials. These products derive from the simultaneous control of both formulations and manufacturing processes, according to the following key points.

- Vitrification is undoubtedly easier for wastes rich in glass forming oxides; the functionalities and consequent usability of the final products, however, are often conditioned by wastes poor in glass forming oxides, but rich in specific oxides, and particularly in iron oxides.
- Engineered formulations allow important reductions of processing times and temperatures, in the transformation of waste‐derived glasses into glass‐ceramics (e.g. considering glasses prone to surface crystallization, in turn leading to sinter‐crystallized glass‐ceramics), or even bring a significant revision of the overall process, leading to glass‐ceramics even without a preliminary vitrification step (direct sintering of wastes, especially if combined with recycled glasses).
- Even if not completely derived from a vitrification step, all the products are associated with an effective stabilization of possible pollutants (in some cases, direct sintering is recommendable to avoid the risk of volatilization of some components during vitrification). The assessment of chemical stability may be seen as an open issue, considering that current efforts are dedicated to the application of conventional leaching tests, but also to the study of the interactions of waste‐derived materials with living cells.^83^
- Glass‐based materials may lead to components with complex combinations of functionalities (as shown for hybrid glass‐based materials), that should no longer be perceived as low‐quality alternatives to ‘standard’ products (from ‘virgin’ raw materials), but something new. The technologies presented here are believed to be only preliminary examples of the potential offered by the transformation of inorganic wastes.
